# The lag-effects of meteorological factors and air pollutants on child respiratory diseases in Fuzhou, China

**DOI:** 10.7189/jogh.12.11010

**Published:** 2022-08-17

**Authors:** Zhengqin Wu, Chong Miao, Haibo Li, Shaowei Wu, Haiyan Gao, Wenjuan Liu, Wei Li, Libo Xu, Guanghua Liu, Yibing Zhu

**Affiliations:** 1 Fujian Obstetrics and Gynecology Hospital, Fuzhou, China; 2 Fujian Maternity and Child Health Hospital, College of Clinical Medicine for Obstetrics & Gynecology and Pediatrics, Fujian Medical University, Fuzhou, China; 3 Fujian Children's Hospital, Fuzhou, China; 4 Fujian Key Laboratory of Women and Children's Critical Disease Research, Fuzhou, China

## Abstract

**Background:**

The effects of meteorological factors and air pollutants on respiratory diseases (RDs) were various in different populations according to the demographic characteristics, and children were considered a vulnerable population. Previous studies were mainly based in cities with serious air pollution. This study aimed to qualify the lag effects of meteorological factors and air pollution on respiratory diseases among children under 18 years old in Fuzhou.

**Methods:**

Meteorological data, air pollutants concentrations and hospital admission data of Fujian Maternity and Child Health Hospital between 2015 and 2019 were collected. A Distributed Lag Nonlinear Model (DLNM) was used to evaluate the nonlinear and lagged effect of meteorological factors and air pollutants on daily RDs number. A subgroup analysis was also conducted to evaluate the effect on different sex groups and age groups.

**Results:**

A total number of 796 125 RDs visits was included during the study period. For meteorological factors, lower mean temperature and relative humidity were significantly associated with daily RDs number (peak relative risk (RR) = 1.032 (95% confidence interval (CI) = 1.011-1.053) and 1.021 (95% CI = 1.013-1.029)), while lower wind speed showed a significant association at low range (peak RR = 0.995 (95% CI = 0.992-0.999)). Temperature warming was a significant protective factor for RDs (peak RR = 0.989 (95% CI = 0.986-0.993)). For air pollutants, SO_2_, NO_2_, PM_10_ and PM_2.5_ were all significantly associated with RDs (peak RR = 1.028 (95% CI = 1.022-1.035), 1.024 (95% CI = 1.013-1.034), 1.036 (95% CI = 1.025-1.047), 1.028 (95% CI = 1.019-1.037)), and the relationship had no threshold. The estimated RR and peak lag day did not change extremely between subgroups.

**Conclusions:**

The findings provide statistical evidence for the prevention of child RDs. In addition, our findings suggested that even at low concentrations, air pollutants still have negative effects on the respiratory system.

Due to the high incidence rate, respiratory diseases (RDs) have become serious threats to people’s health, especially children. According to the statistics, over four million people die due to RDs each year [1]. As the susceptible population, nearly three million children die of pneumonia and low respiratory tract infections [2]. The causes of RDs are complicated, despite pathogen infection, environmental factors are also considered to be important factors affecting the incidence of RDs. Among environmental factors, meteorological factors and air pollution have the longest inference time and the largest inference range, thus, having a huge impact on RDs. Climate change may cause physiological changes, increase physiological stress, and further cause respiratory symptoms, moreover, it also has an impact on allergic RDs [3]. Previous studies have pointed out that children are more vulnerable to air pollution and meteorological factors since they may more likely to be affected by extreme climates due to their higher surface area to body weight ratios and lower immunity than adults [4]. The vulnerability to meteorological factors and air pollutants was various in different populations according to the demographic characteristics [5], therefore, it is necessary to conduct independent studies for each population in each region. Previous studies on air pollution and child RDs are mainly based in cities with severe air pollution, lack of research on the impact of low-concentration air pollutants. However, many studies have found that the effects of air pollutant concentrations on health may have no threshold, meaning even at a very low concentration, air pollutants still have the potential effects on health [6-8]. This article was based in Fuzhou, a city with high air quality, focused on children under 18 years old, and aimed to qualify the lag effects of meteorological factors and air pollution on child RDs.

## METHODS

### Daily RDs number calculation

This study was a hospital-based retrospective study. The original data came from Fujian Maternity and Child Health Hospital. The daily RDs number was calculated from outpatient visits due to respiratory diseases, which was extracted from the hospital electronic medical record system and patient information system. Considering the integrity of the information and the impact of the quarantine policy due to coronavirus disease 2019, the study period was from January 2015 to December 2019. The extracted data included the outpatient visit date, sex, age and main diagnosis, and patients with missing diagnosis data will be excluded from the study. The clinical information of respiratory patients was filtered according to the International Classification of Diseases, 10th edition (ICD-10) codes and further calculated to daily RDs number. The selected ICD-10 codes were respiratory diseases (J00-J99), to avoid omissions, respiratory clinical symptoms (R04-R06) were also included. The data were grouped by sex and age for further subgroup analysis.

### Meteorological factors and air pollution data

The meteorological data were collected from National Meteorological Information Centre. The daily mean value, the highest value, the lowest value of temperature, relative humidity (RH) and wind speed was recorded at the observatory station in Fuzhou (Station number:58847). The air pollution data was collected from the Division of Atmospheric Environment, Fujian Provincial Department of Ecology and Environment, which contained daily mean concentrations of sulphur dioxide (SO_2_), nitrogen dioxide (NO_2_), particulate matter smaller than 2.5 μm (PM_2.5_) and particulate matter smaller than 10 μm (PM_10_). The collection period was from January 2015 to December 2019.

### Statistical methods

All statistical analyses were conducted by software R (version 4.1.0), and a *P*-value <0.05 was considered statistically significant in this study. First, Spearman correlation coefficients between daily RDs number, meteorological factors and air pollutants were calculated in order to evaluate the association between valuables. The dose-response relationship between meteorological factors, air pollutants and RDs were considered nonlinear [9], thus, we used distributed lag nonlinear models (DLNMs) [10] to evaluate the nonlinear and lagged effect of meteorological factors and air pollutants on daily RDs number. For meteorological variables, the maximum lag days were set to 7, and for air pollutant variables, the maximum lag days were set to 30. To evaluate the lag effect of weather factors, we considered air pollutants as confounders, thus, the DLNM model we used is shown below:

log *E* (*Y_t_*) = *cb* (*Z_m_*) + *DOW* + *PubH* + *ns* (*Times*, *8 df/y*) + *ns* (*SO_2_*, *3 df*) + *ns* (*NO_2_*, *2 df*) + *ns* (*PM_10_*, *3 df*) + *ns* (*PM_2.5_*, *2 df*).

where *Y_t_* is the expected daily RDs number on day t; *Z_m_* is each meteorological variable on day t; *cb* is the cross-basis function; *DOW* is the day of the week and *PubH* is the public holiday used to control the impact of hospital outpatient working days; *ns* is the natural spline function used to control the confounders; *Times* is the time variable used to control the impact of time trend; *SO_2_, NO_2_, PM_10_ and PM_2.5_* are air pollutant variables as confounders; *df* is the degree of freedom for each variable used in the model.

To evaluate the lag effect of air pollutants, the DLNM model we used the modification below:

log *E* (*Y_t_*) = *cb* (*Z_a_*) + *DOW* + *PubH* + *ns* (*Times*, *8 df/y*) + *ns* (*TEMP*, *2 df*) + *ns* (*RH*, *2 df*) + *ns* (*WS*, *3 df*) + *ns* (*TC*, *2 df*).

where *Z_a_* is each air pollutants variable, and meteorological variables are now the confounders: *TEMP* is the mean temperature; *RH* is the relative humidity, *WS* is the mean wind speed, and *TC* is the temperature change compared to the last day. The degree of freedom for each variable included in the model was selected by calculating the lowest sum of residuals using the partial autocorrelation function (PACF) and the lowest Akaike information criterion (AIC). In order to avoid potential multicollinearity, according to the results of the correlation test, when evaluating a certain variable, confounders with Spearman correlation coefficients greater than 0.4 (*P* < 0.05) will be removed from the model. We used the generalized variance inflation factor (GVIF) to evaluate the potential multicollinearity for each model after the model was established.

After the model was established, a calculating value and reference value for each variable was set to evaluate the relative risk (RR) and the peak lag effect day. Mean values were used as the reference value for mean temperature, RH and wind speed, and 25^th^ percentile (Q_1_) and 75^th^ percentile (Q_3_) were used to calculate the RR for each lag day. For temperature change, 0 was used as the reference value, and the 50^th^ percentile (Q_2_) for both cooling and warming was used as calculating values. For air pollutant variables, 0 was used as the reference value, and the interquartile range (IQR) for each value was used as incremental calculating values. A subgroup analysis was conducted to evaluate the difference of the lag effect in different subgroups.

A sensitivity analysis was conducted to test the validity of the model, by changing the *df* value for each variable from 0-10 for each value (for *Times*, *df* was changed from 0-10/y). The change of *df* did not change the lag effect and its significance, and did not change the peak lag day of the lag effect.

## RESULTS

### Baseline analysis

**Figure 1:**
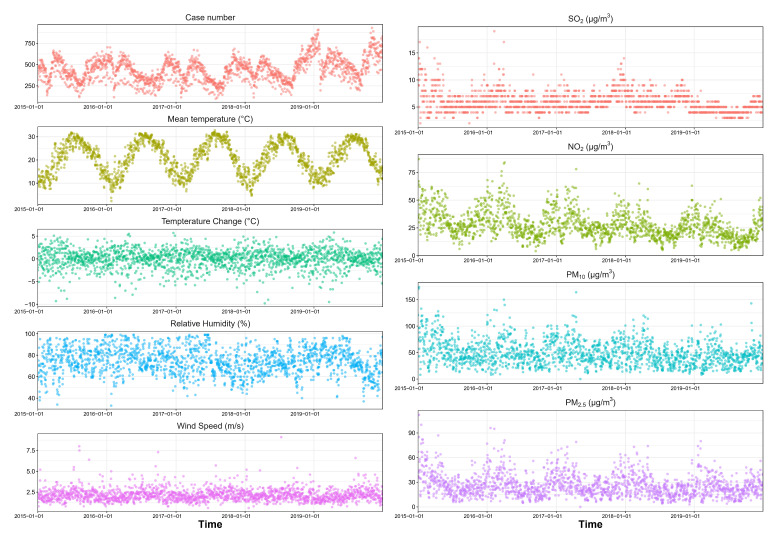
Time series distribution of daily number of outpatient visit for respiratory diseases, meteorological factors and air pollutants.

The distribution of diseases was shown in **Table 1** and the statistical summary of each meteorological factor and air pollution data was shown in **Table 2**. The time-series distribution of daily RDs number, meteorological factors and air pollutants were shown in **Figure 1**. After the data extraction and cleaning, 964 012 respiratory disease clinical records were extracted and organized into grouped daily RDs data. From January 2015 to December 2019, the total number of visits for RDs is 796 125. Among them, 480 711 (60.38%) were male, 315 357 (39.61%) were female. After grouped by age, 485 701 (61.01%) from 0-3 years old, 239 331 (30.06%) from 3-6 years old, and 71 079 (8.93%) from 6-18 years old. The number of male child RDs number is higher than females, suggesting that male children may be more susceptible to respiratory diseases than female children. The time-series figure suggested that during the period of low temperature (autumn and winter), the number of outpatient visits for the respiratory system has a trend of increasing. The mean daily concentration was 5.93μg/m^3^ for SO_2_, 27.60μg/m^3^ for NO_2_, 49.08μg/m^3^ for PM_10_, 26.17μg/m^3^ for PM_2.5_. For meteorological factors, the daily mean temperature was 20.94°C, the daily mean RH was 74.43% and the daily mean wind speed was 2.17m/s. All clinical data, meteorological data and air pollution data had valid sources and the missing data was less than 0.1%.

**Table 1:** Distribution of respiratory-related outpatient visits of Fujian Maternity and Child Health Hospital from 2015 to 2019

Category	ICD-10	Number of visits (proportion)
Acute upper respiratory infections	J00-J06	97 258 (12.21%)
Influenza and pneumonia	J09-J18	148 798 (18.69%)
Other acute lower respiratory infections	J20-J22	207 740 (26.09%)
Other diseases of upper respiratory tract	J30-J39	51 709 (6.50%)
Chronic lower respiratory diseases	J40-J47	240 822 (30.25%)
Other diseases of the respiratory system	J60-J99	29 457 (3.70%)
Respiratory clinical symptoms	R04-R06	20 341 (2.56%)
Total		796 125

**Table 2:** Statistical summary of daily number of outpatient visits for respiratory diseases, meteorological factors and air pollutants

Variable	Mean	SD	Min	Q_1_	Median	Q_3_	Max	IQR
**Daily RDs number**	436.23	138.19	99	331	436	528	931	197
**Daily RDs number (male)**	263.40	82.27	60	201	263	319	571	118
**Daily RDs number (female)**	172.80	57.70	30	129	170	209	370	80
**Daily RDs number (0-3 y)**	266.13	78.70	57	205	264	321	548	116
**Daily RDs number (3-6 y)**	131.14	57.98	10	87	127	167	384	80
**Daily RDs number (6-18 y)**	38.95	19.72	2	25	35	49	134	24
**Mean temperature (°C)**	20.94	6.83	2.3	15.1	21.5	27	32.8	11.9
**Temperature change (°C)**	-0.001	2.05	-9.8	-1.1	0.2	1.3	5.8	2.4
**Relative humidity (%)**	74.43	12.05	33	66	75	83	99	17
**Wind speed (m/s)**	2.17	0.75	0.6	1.7	2.1	2.5	9.1	0.8
**SO_2_ (μg/m^3^)**	5.93	1.86	2	5	6	7	19	2
**NO_2_ (μg/m^3^)**	27.60	12.01	4	19	25	34	87	15
**PM_10_ (μg/m^3^)**	49.08	23.20	8	33	45	62	174	29
**PM_2.5_ (μg/m^3^)**	26.17	13.84	3	17	24	32	112	15

RDs – respiratory diseases, SO_2_ – sulphur dioxide, NO_2_ – nitrogen dioxide, PM_10_ – particulate matter smaller than 10 μm, PM_2.5_ – particulate matter smaller than 2.5 μm, Min – minimal value, Q_1_ – 25^th^ percentile, Q_3_ – 75^th^ percentile, Max – maximal value, IQR – interquartile range

The Spearman correlation coefficient between daily RDs numbers, meteorological factors and air pollutants were shown in **Table 3**. As mentioned before, we exclude confounders with high correlation to avoid potential multicollinearity. For example, when calculating the effect of RH, we exclude SO_2_ from the model, as it showed a significantly high correlation coefficient with RH. No model showed serious multicollinearity according to the GVIFs calculated after the models were established.

**Table 3:** Spearman correlation coefficients between meteorological factors and air pollutants

	TEMP	TC	RH	WS	SO_2_	NO_2_	PM_10_	PM_2.5_
**TEMP**	1							
**TC**	0.12*	1						
**RH**	-0.03	-0.11*	1					
**WS**	0.19*	-0.16*	-0.32*	1				
**SO_2_**	-0.11*	0.21*	-0.46*	-0.04	1			
**NO_2_**	-0.39*	0.24*	0.23*	-0.46*	0.41*	1		
**PM_10_**	-0.04	0.27*	-0.36*	-0.18*	0.63*	0.52*	1	
**PM_2.5_**	-0.26*	0.2*	-0.21*	-0.26*	0.54*	0.58*	0.89*	1

TEMP – mean temperature, TC – temperature change, RH – relative humidity, WS – wind speed, SO_2_ – sulphur dioxide, NO_2_ – nitrogen dioxide, PM_10_ – particulate matter smaller than 10 μm, PM_2.5_ – particulate matter smaller than 2.5 μm

*P < 0.05.

### Association between meteorological factors and daily RDs number

**Figure 2:**
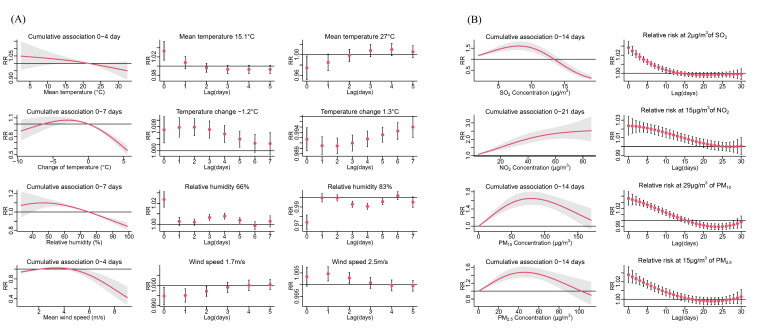
Cumulative association and relative risk with different lag days of meteorological factors and air pollutants on respiratory diseases outpatient visit number. **Panel A.** Meteorological factors. **Panel B.** Air pollutants.

**Figure 2**, panel A showed the estimated cumulative association and the lag time effect of meteorological factors on daily RDs number. The left figure showed the cumulative association between daily RDs number and meteorological factors, and the right figure showed the lag time effect of calculating value on daily RDs number. There was a negative association between mean temperature and daily RDs number, compared to the reference value, lower mean temperature (Q_1_) had a higher estimated RR on RDs at the peak lag day (day 0, RR = 1.032 (95% CI = 1.011-1.053)), and higher mean temperature (Q_3_) had a lower estimated at the peak lag day (day 0, RR = 0.976 (95% CI = 0.953-0.999). A nonlinear negative association was found between RH and RDs number, compared to the reference value, lower RH (Q_1_) had a higher estimated RR on RDs at the peak lag day (day 0, RR = 1.021 (95% CI = 1.013-1.029)), higher RH (Q_3_) had a lower estimated RR on RDs at the peak lag day (day 0, RR = 0.975 (95% CI = 0.967-0.983)). A nonlinear association was found between wind speed and RDs number, compared to the reference value, lower wind speed (Q_1_) had a lower estimated RR on RDs at the peak lag day (day 1, RR = 0.995 (95% CI = 0.992-0.999)), higher wind speed (Q_3_) had a higher estimated RR on RDs at the peak lag day (day 1, RR = 1.004 (95% CI = 1.001-1.007)). For temperature change, compared to the reference value, decreased temperature compared to the last day (-1.3°C) had a higher estimated RR on RDs at the peak lag day (day 2, RR = 1.007 (95% CI = 1.004-1.010)), and increased temperature (1.2°C) had a lower estimated RR on RDs at the peak lag day (day 2, RR = 0.989 (95% CI = 0.986-0.993)), for all meteorological factors, the RR was approximately 1 after lag day 7.

### Association between air pollutants and daily RDs number

**Figure 3:**
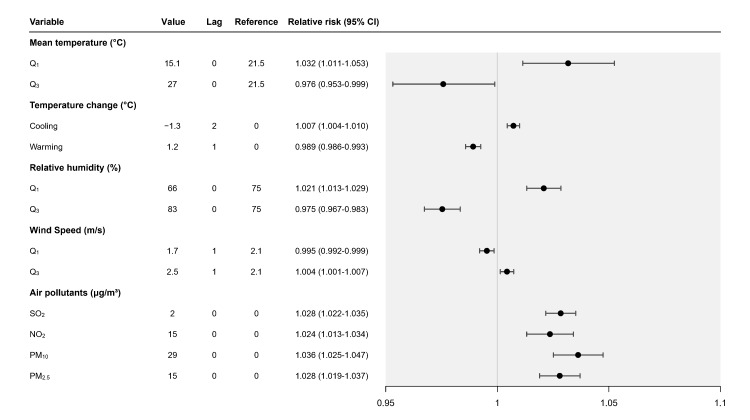
Most significant relative risk and lag day of meteorological factors and air pollutants. Value – the value of variables used to estimate relative risk, Relative risk – highest relative risk in lag days, CI – confidence interval, Lag – the day that highest relative risk appears, Reference – the value of variables as reference value, SO_2_ – sulphur dioxide, NO_2_ – nitrogen dioxide, PM_10_ – particulate matter smaller than 10 μm, PM_2.5_ – particulate matter smaller than 2.5 μm, Q_1_ – 25^th^ percentile, Q_3_ – 75^th^ percentile.

**Figure 2**, panel B showed the estimated cumulative association and the lag time effect of air pollutants on daily RDs number. The left figure showed the cumulative association between air pollutants and RDs number, and the right figure showed the lag time effect of calculating value on RDs number. All air pollutants showed a positive nonlinear association with RDs number, and the peak lag day for all air pollutants was day0. For NO_2_, an increment of 2μg/m^3^ had a higher estimated RR on RDs at the peak lag day (day 0, RR = 1.028 (95% CI = 1.022-1.035)). For SO_2_, an increment of 15μg/m^3^ had a higher estimated RR on RDs at the peak lag day (day 0, RR = 1.024 (95% CI = 1.013-1.034)). For PM_10_, an increment of 29μg/m^3^ had a higher estimated RR on RDs at the peak lag day (day 0, RR = 1.036 (95% CI = 1.025-1.047)). For PM_2.5_, an increment of 15μg/m^3^ had a higher estimated RR on RDs at the peak lag day (day 0, RR = 1.028 (95% CI = 1.019-1.037)). For all air pollutant variables, the RR was approximately 1 after lag day 21. The summary of the lag time effect was shown in **Figure 3**.

### Subgroup analysis

The subgroup analysis of the lag time effect was shown in Table S1 in the **Online Supplementary Document**. The relationship between meteorological factors, air pollutants and daily RDs in different subgroups was similar to the overall effect. The estimated RR and peak lag day did not change extremely between different groups.

## DISCUSSION

In general, we estimated the lag time effect of meteorological factors and air pollutants on child RDs. For meteorological factors, mean temperature and RH was negatively associated with daily RDs number, while wind speed showed a positive association in the low range. Temperature warming was a protective factor for RDs. All air pollutants were positively associated with RDs, and the effect showed no threshold in our study.

Meteorological factors and air pollutants are closely related to human health. People have long been aware of the impact of meteorological factors and air pollution on health [11], and the respiratory system is more directly and significantly affected [12]. The effect is different in cohorts with different ages, genders, and races [13]. These demographic characteristics are not the same in different regions. Fuzhou is one of the hottest cities in China. Affected by the East Asian monsoon, the summer in Fuzhou is hot and humid, and extremely high temperature and severe temperature changes are prone to occur, thus, could be a suitable city to study the effects of meteorological factors on RDs. On the other hand, the air pollution in Fuzhou is not serious, thus it can be a suitable platform to explore the health effects of air pollution at a very low concentration. This study was based on the hospital clinical data from Fujian Maternity and Child Health Hospital, the largest maternal and child medical centre in Fujian province, the daily RDs patient number can better reflect the incidence of respiratory diseases in children.

We estimated the potential correlation between daily RDs, meteorological factors and air pollutants. As the result, there was a strong negative correlation between RH and SO_2_, as well as wind speed and NO_2_. These findings were consistent with previous research [14,15]. Thus, we exclude these confounders to avoid potential multicollinearity.

Lower temperature was considered associated with the incidence of asthma and respiratory tract infections (RTIs) [16]. In our study, we found that temperature has a negative association with RDs. Previous studies have pointed out that the effect of temperature may be U-shaped, but in our study, the relationship was monotonically negative. The positive association between lower temperature and RDs was consistent with the previous study [17], however, we observed a protective effect of high temperature on RDs, which contract to the previous study which pointed out that the incidence of RDs among children was increasing during the heatwave [18-20]. This contract may be due to the general use of air conditioning in hot weather, which reduces the potential effect of high temperature. Extreme temperature change is also a potential inference factor of RDs. Most previous studies used diurnal temperature range (DTR) to evaluate the temperature change, however, Fuzhou has a subtropical monsoon climate, and the temperature in Fuzhou is greatly affected by rainfall. This characteristic may lead to a sudden temperature change between two days when rainfall occurs which DTR cannot perfectly reflect. Moreover, DTR can only reflect the absolute value of temperature change, but cannot reflect the increasing or decreasing temperature. Thus, we used the variance of mean temperature between two days to evaluate the effect of temperature change on RDs. We found a positive association between temperature change and daily RDs, which is consistent with the findings of previous studies [21]. A study pointed out that rapid temperature change can activate more eosinophils and further lead to a more severe inflammatory response [22]. The rapid temperature change was also considered positively associated with asthma and RTIs, especially for children and elders [23]. RH was found to have a protective effect on RDs in this study, which was consistent with another study conducted in Seoul [24] and Busan [25], Korea. Lower relative humidity favours the aerosol transmission of viruses, such as influenza [26], which will further lead to RDs. On the other hand, some studies have pointed out that high relative humidity can increase the incidence of allergic diseases, including asthma and other diseases [27]. These mechanisms lead to the inconsistency of the overall effect of RH on RDs in different studies [28]. We found the daily mean wind speed had a protective effect on RDs at the low-to-moderate range, which was shown in **Figure 2**, panel A.The effect may be due to the increase in wind speed increases the thermal evaporation loss from the body temperature [29], which further leads to the incidence of RDs. However, the effect of wind speed was negative on extreme high value, this may be due to a decrease in people's willingness to visit hospitals for mild RDSs during inclement weather. All meteorological factors showed a short-term effect.

The concentration of multiple air pollutants was associated with meteorological factors, thus, when evaluating the effect of air pollutants, we control the potential effect of meteorological factors. All the air pollutants included in this study had a positive association with daily RDs, and the association was nonlinear and non-threshold, which supported the theory we mentioned above. For PM_10_ and PM_2.5_, the effects tend to decrease at a high concentration level; moreover, for SO_2_, the lag effect showed an unexpected negative at high concentrations. This unexpected result was mainly due to the insufficient number of sample days with high SO_2_ concentration. During the study period, only 44 days had SO_2_ concentrations ≥10μg/m^3^, of which only 8 days had SO_2_ concentrations ≥14μg/m^3^, we did not have enough days with high-level concentration to evaluate the effect. This makes the model vulnerable to extreme data, thus the model was not reliable at higher SO_2_ concentrations (≥10μg/m^3^), which further led to this unexpected result. Among all the air pollutants, PM_10_ had the highest RR with an increment of IQR. Another study that studies the effect of air pollutants on respiratory diseases on the whole population in Fuzhou suggested that the PM_10_ and PM_2.5_ were not significant [30]. The contract suggested that children under 18 years old maybe more susceptible to PM_10_ and PM_2.5_. Our findings are consistent with the previous conjecture that even at low concentration, SO_2_, NO_2_, PM_10_ and PM_2.5_ was harmful to health. These findings suggested that even in areas with relatively good air quality, reducing air pollution can reduce the incidence of child RDs.

The results of the subgroup analysis showed that there were no significant differences in the lag effect of the meteorological factors and air pollution of children in different age groups under the age of 18. The significance of some effects vanished in high-age groups (3-6 years old and >6 years old) may be due to the lack of sample number, and the peak lag day did not change extremely. As shown in **Figure 2**, the impact of meteorological factors on RDs showed a short-term effect, and the effect vanished within 7 days; while the impact of air pollution on RDs showed a long-term effect, which lasted for 2-4 weeks. The findings help us take measures to prevent RDs. For example, taking immediate precautions such as warming and humidification can help protect children from RDs due to extreme weather. On the other hand, air pollution requires long-term and continuous control. Moreover, even in cities with high air quality, reducing air pollution still has public health significance.

This study had several strengths and limitations. First of all, this is the first study about the effect of meteorological factors and air pollutants on RDs which focus on the population under 18 years old in Fuzhou. As mentioned before, Fuzhou has the clinical characteristic of rapid temperature change, which is suitable as a city for studying the influence of meteorological factors on RDs. Moreover, we could also estimate the effect of air pollutants at the low-concentration level. Secondary, the study was conducted based on the clinical data of Fujian Maternity and Child Health Hospital, the largest maternity and child centre in Fujian. The amount of data used for research can be guaranteed, and the daily outpatient volume can better reflect the incidence of child RDs in Fuzhou. The data used in this study were all collected from government agencies, authoritative data centres and hospital clinical data systems to ensure reliability and had a low missing rate. On the other hand, the study still had some limitations. Considering the air quality in Fuzhou, we did not have enough sample days with severe air pollution to evaluate the effect of high-level air pollution on RDs, the estimated value may be easily affected by minor days, as the result, the effect value at high concentration tended to reduce, especially for the SO_2_ model. Secondary, according to the age distribution of RDs patients in the hospital, the insufficient number of patients over the age of six prevented us from making a finer grouping of RDs numbers. As the result, we could not estimate the difference of effect on elder children (>12 years old). Finally, we used daily RDs numbers to reflect the incidence of RDs, this may cause potential selection bias since the hospital is located in the city centre, therefore, the generalizability of the conclusions of this study was limited and may not apply to all children, especially those in rural areas.

## CONCLUSIONS

We used DLNM to evaluate the lagged effect of meteorological factors and air pollutants on daily RDs number under 18 years old. As the result, mean temperature, temperature change and RH were negatively associated with daily RDs number, while wind speed was positively associated at the low range. For all air pollutants included in the study, NO_2_, SO_2_, PM_10_ and PM_2.5_, were all positively associated with daily RDs number, and the relationship was nonlinear and non-threshold. The effect of meteorological factors was the short-term effect, while the influence of air pollutants was long-lasted. These effects did not differ much across age groups defined in this study, a future study based on the data set with more average age distribution would provide a more comprehensive conclusion about the effect of meteorological factors and air pollutants on elder children.

## Additional material


Online Supplementary Document
