# Supplementary material for: The lag-effects of meteorological factors and air pollutants on child respiratory diseases in Fuzhou, China
Source: J Glob Health. 2022 Aug 17;12:11010. doi: 10.7189/jogh.12.11010 (PMC9380967; doi:10.7189/jogh.12.11010)
Supplement: Online Supplementary Document [file jogh-12-11010-s001.pdf]

Table S1. Group-stratified most significant relative risk and lag day of meteorological factors and air pollutants.

Variable	Value	Male		Female		0-3year		3-6year		>6year	
		**Lag**	**RR** [95% CI]	**Lag**	**RR** [95% CI]	**Lag**	**RR** [95% CI]	**Lag**	**RR** [95% CI]	**Lag**	**RR** [95% CI]
**Mean temperature (°C)**											
Q_1	15.1	0	1.030 [1.009; 1.051]	0	1.038 [1.015; 1.061]	0	1.034 [1.015; 1.054]	0	1.037 [1.007; 1.067]	0	1.023 [0.979; 1.070]
Q_3	27	0	0.981 [0.957; 1.004]	0	0.965 [0.940; 0.991]	0	0.966 [0.945; 0.989]	0	0.978 [0.946; 1.012]	6	0.978 [0.944; 1.013]
**Temperature change (°C)**											
Cooling	-1.3	2	1.007 [1.004; 1.010]	2	1.009 [1.006; 1.012]	2	1.011 [1.008; 1.014]	0	1.002 [0.998; 1.006]	0	1.006 [0.996; 1.016]
Warming	1.2	2	0.990 [0.987; 0.993]	2	0.987 [0.984; 0.990]	2	0.986 [0.984; 0.989]	0	0.993 [0.989; 0.998]	1	0.990 [0.982; 0.997]
**Relative humidity (%)**											
Q_1	66	0	1.023 [1.015; 1.031]	0	1.025 [1.016; 1.033]	0	1.026 [1.019; 1.033]	0	1.018 [1.008; 1.029]	0	1.025 [1.008; 1.042]
Q_3	83	0	0.974 [0.966; 0.982]	0	0.973 [0.964; 0.981]	0	0.972 [0.964; 0.979]	0	0.979 [0.968; 0.991]	0	0.965 [0.947; 0.983]
**Wind Speed (m/s)**											
Q_1	1.7	0	0.995 [0.990; 0.999]	0	0.995 [0.990; 0.999]	1	0.993 [0.990; 0.996]	0	0.995 [0.988; 1.001]	6	1.006 [0.999; 1.013]
Q_3	2.5	1	1.005 [1.002; 1.008]	1	1.004 [1.001; 1.008]	1	1.006 [1.003; 1.009]	0	1.004 [0.999; 1.008]	6	0.994 [0.988; 1.001]
**Air pollutants (μg/m³)**											
SO_2	2	0	1.030 [1.023; 1.037]	0	1.026 [1.014; 1.038]	0	1.021 [1.015; 1.027]	0	1.040 [1.030; 1.051]	0	1.050 [1.034; 1.066]
NO_2	15	1	1.022 [1.014; 1.031]	0	1.026 [1.019; 1.034]	1	1.014 [1.006; 1.022]	3	1.038 [1.029; 1.046]	0	1.057 [1.034; 1.081]
PM_{10}	29	0	1.033 [1.021; 1.044]	0	1.041 [1.029; 1.054]	0	1.024 [1.014; 1.035]	0	1.020 [1.012; 1.029]	0	1.061 [1.035; 1.088]
$PM_{2.5}$	15	0	1.024 [1.015; 1.033]	0	1.034 [1.024; 1.044]	0	1.020 [1.012; 1.029]	0	1.055 [1.038; 1.072]	0	1.039 [1.019; 1.060]

Value – the value of variables used to estimate relative risk, RR – highest relative risk in lag days, CI – confidence interval, Lag – the day that highest relative risk appears, SO_2 – sulfur dioxide, NO_2 – nitrogen dioxide, PM_{10} – particulate matter smaller than 10 μm, $PM_{2.5}$ – particulate matter smaller than 2.5 μm, Q_1:25th quantiles, Q_3 – 75th quantiles.
